# High-Index-Faceted Cu Nanoparticles in Ag–Cu Tandem Catalysts for Selective CO_2_ Electroreduction

**DOI:** 10.1021/acsnanoscienceau.6c00129

**Published:** 2026-09-07

**Authors:** Guangtao Wang, Simin Li, Chao Yu, Yang Zhou, Wenlei Zhu, Bo Shen

**Affiliations:** † Department of Materials Science and Engineering, 53025 City University of Hong Kong, Hong Kong, China; ‡ State Key Laboratory of Water Pollution Control and Green Resource Recycling, State Key Laboratory of Analytical Chemistry for Life Science, Frontiers Science Center for Critical Earth Material Cycling, School of Environment, School of Chemistry and Chemical Engineering, 12581 Nanjing University, Nanjing 210023, China; § School of Environmental and Chemical Engineering, 12676 Jiangsu University of Science and Technology, Zhenjiang 212100, China; ∥ Research Institute of Tsinghua University in Shenzhen, Shenzhen 518057, China; ⊥ State Key Laboratory of Flexible Electronics (LoFE), Institute of Advanced Materials (IAM), 12577 Nanjing University of Posts and Telecommunications (NJUPT), Nanjing 210023, China

**Keywords:** Ag−Cu catalysts, high-index facets, nanoparticles, CO_2_ electroreduction, ethylene

## Abstract

Facet engineering of Cu-based nanocatalysts is a promising strategy for regulating the selectivity of CO_2_ and CO electroreduction because surface atomic arrangements strongly influence intermediate adsorption and reaction pathways. Compared with low-index facets, high-index facets contain abundant steps, kinks, and low-coordinated atoms, providing diverse active ensembles that can facilitate CO adsorption and subsequent C–C coupling. However, the catalytic roles of high-index-faceted Cu nanocatalysts remain largely unexplored. Herein, tetrahexahedral (THH) and hexoctahedral (HOH) high-index-faceted Cu nanoparticles were integrated with a porous Ag foam to construct Cu/Ag tandem catalysts for CO_2_ electroreduction. The C_2_^+^ selectivity follows the order HOH Cu/Ag > THH Cu/Ag > non-facet-controlled Cu/Ag, with HOH Cu/Ag achieving the highest ethylene Faradaic efficiency of 60.37%. In situ spectroscopic measurements using HOH Cu/Ag as a representative high-index-faceted catalyst indicate the formation of abundant adsorbed CO and possible CO-derived coupling intermediates within the potential range associated with pronounced ethylene production. Stability measurements further show that the morphology and catalytic selectivity are largely retained under moderate operating conditions, whereas more negative potentials induce surface roughening and gradual performance decay. This work provides a platform for investigating morphology-dependent selectivity in high-index-faceted Cu catalysts during CO_2_ electroreduction.

## Graphical abstract

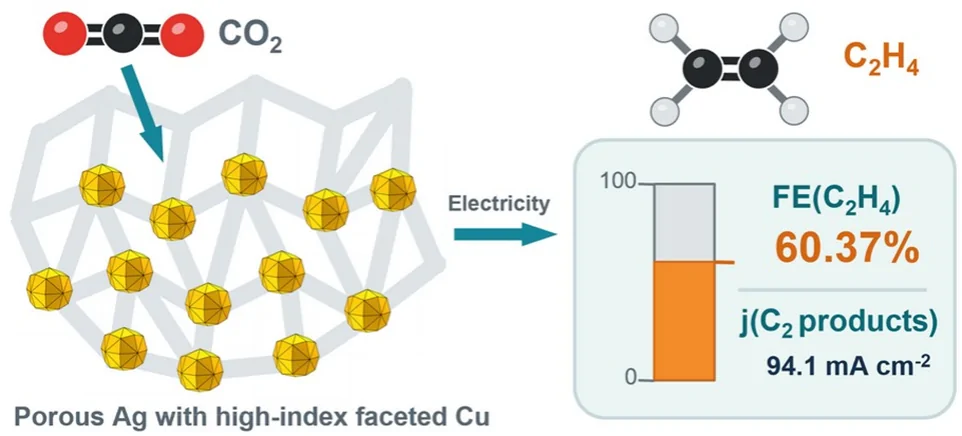

## 1 Introduction

Electrochemical CO_2_ reduction provides a sustainable route for converting CO_2_ into fuels and value-added chemicals using renewable electricity.^1–3^ Among the various electrocatalysts investigated, Cu is uniquely capable of promoting C–C bond formation and producing a broad range of multicarbon products.^4–6^ However, the multiple competing reaction pathways on Cu often result in broad product distributions and limited selectivity toward a desired multicarbon product.^7,8^ One strategy to promote multicarbon formation is to increase the availability of CO-derived intermediates at Cu surfaces.^9–11^ Cu/Ag tandem catalysts have therefore attracted considerable attention because they combine the complementary catalytic properties of the two metals: Ag selectively promotes CO formation, whereas Cu facilitates the further conversion of CO into multicarbon products.^12–16^ To date, Cu/Ag tandem catalysts have mainly been optimized by tuning the catalyst composition, particle size, and spatial distribution.^17–19^ Despite these advances, the formation and branching of multicarbon products still occur on the Cu component, making the atomic structure of the exposed Cu surface a key factor in determining tandem catalytic selectivity.^20–26^

Engineering the exposed surface of Cu nanoparticles through morphology control has emerged as an effective strategy for tuning CO_2_ electroreduction selectivity.^27^ Extensive studies using Cu single crystals, shape-controlled nanoparticles, and theoretical models have shown that changes in surface atomic arrangement can lead to different reaction pathways and product distributions.^28–31^ For example, Cu(100)-type motifs are frequently associated with adsorption configurations favorable for CO-derived intermediate coupling and C_2+_ product formation, whereas Cu(111)-type surfaces exhibit different CO binding and protonation behaviors.^28,31,32^ Compared with the relatively well-studied low-index facets, high-index-faceted Cu nanoparticles contain ordered combinations of terraces, steps, kinks, and low-coordinated atoms, providing more diverse local atomic arrangements and reaction environments. Such structural complexity offers additional opportunities to regulate product distribution beyond conventional low-index Cu surfaces.^33–35^ However, such nanostructures remain underexplored because well-defined high-index-faceted Cu nanoparticles are difficult to synthesize. This limitation is particularly evident in Cu/Ag tandem catalysts, where the exposed Cu surface has rarely been deliberately controlled as an independent structural variable. As a result, the contribution of high-index-faceted morphology to tandem catalytic selectivity is still poorly understood.

Herein, we use porous Ag foam as a common tandem platform and introduce tetrahexahedral (THH) and hexoctahedral (HOH) Cu nanoparticles as the principal structural variable for tandem CO_2_ electroreduction (Figure 1). These two characteristic morphologies are generally associated with {*hk*0}-type and {*hkl*}-type facets, respectively.^36–38^ By minimizing variations in catalyst composition and tandem architecture, this design allows the influence of Cu surface structure on tandem CO_2_ electroreduction to be evaluated under comparable conditions. Both high-index-faceted Cu/Ag tandem catalysts exhibit higher ethylene selectivity than the non-facet-controlled Cu/Ag catalyst, with ethylene Faradaic efficiency (FE) of 53.34% for THH Cu/Ag and 60.37% for HOH Cu/Ag. In situ spectroscopic characterization provides insight into the adsorption and coupling of CO-derived intermediates over representative high-index-faceted Cu/Ag catalysts, while stability tests show that the characteristic Cu nanoparticle morphologies can be largely retained under moderate electrolysis conditions. These findings demonstrate the important role of high-index-faceted Cu nanoparticle morphology in regulating product selectivity during tandem CO_2_ electroreduction and provide guidance for the rational design of Cu/Ag tandem catalysts.

**Figure 1:**
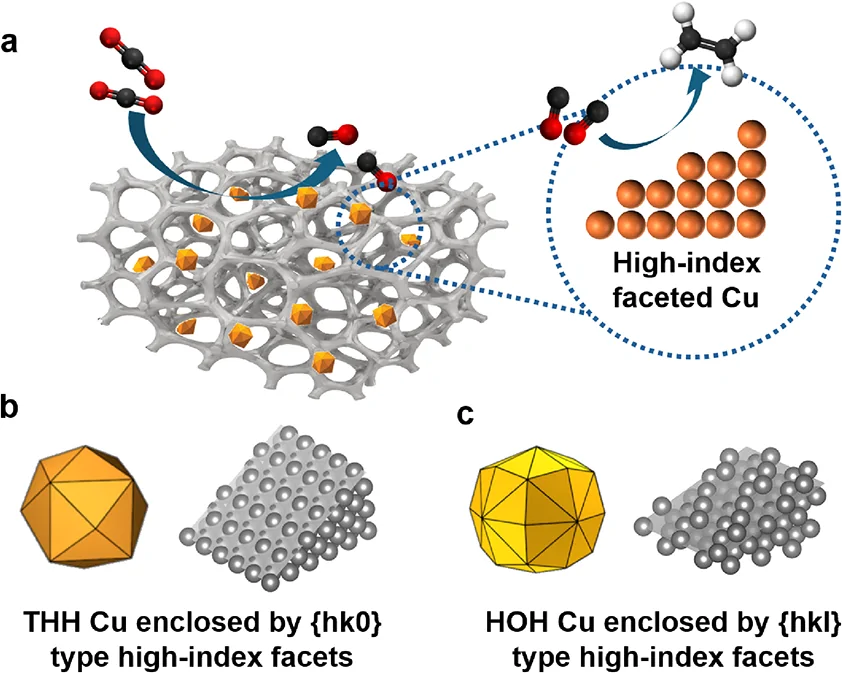
Schematic illustration of the high-index-faceted Cu/Ag tandem catalyst. (a) High-index-faceted Cu nanoparticles supported on porous Ag foam for tandem CO_2_ electroreduction. The enlarged view shows a schematic high-index Cu surface containing low-coordination surface sites. Geometrical models of (b) THH Cu and (c) HOH Cu nanoparticles, representing two characteristic high-index-faceted Cu morphologies investigated in this work.

## 2 Materials and Methods

### 2.1 Chemicals and Materials

Copper­(II) nitrate hydrate (Cu­(NO_3_)_2_·2.5H_2_O; ≥99.99%), potassium bicarbonate (KHCO_3_, 99.7%) and telluric acid (H_6_TeO_6_, purity 98%) were purchased from Macklin. Ethanol (CH_3_CH_2_OH, 99.9%), dimethyl sulfoxide (DMSO, 99.9%), and deuterium oxide (D_2_O, 99.9 atom %) were purchased from Aladdin. Concentrated nitric acid (HNO_3_, 68 wt %) and fuming nitric acid (HNO_3_, 98 wt %) were purchased from Hushi (Shanghai, China). A proton exchange membrane (Nafion 212, Self-supporting, DuPont) was purchased from SCI Materials Hub. Commercial silver foam was purchased from Kunshan Yushan Town Jiayisheng New Materials Business Department (Kunshan, Jiangsu, China). All chemical reagents were used as received without any further purification. Deionized water (18.2 MΩ·cm) was used in all experimental processes.

### 2.2 Synthesis of High-Index-Faceted Cu/Ag Tandem Catalysts

Commercial Ag foam sheets with a nominal thickness of 200 μm were cut into 2.0 × 2.0 cm^2^ pieces, sequentially rinsed with deionized water and ethanol, and dried before use. The precursor ink consisted of 173 μL of a Cu­(NO_3_)_2_·2.5H_2_O solution (150 mg mL^–1^) and 85.5 μL of a telluric acid solution (10 mg mL^–1^ in water), corresponding to a Cu/Te molar ratio of approximately 30:1. Concentrated HNO_3_ (10 μL) was added to facilitate complete dissolution of the precursors. During spray deposition, a mask was used to define a central 1.0 × 1.0 cm^2^ coating area corresponding to the region exposed to the flow channel during electrochemical measurements. The precursor solution was then uniformly sprayed onto the exposed area on both sides of the Ag foam from a distance of approximately 2 cm while the substrate was maintained at 60 °C to facilitate solvent evaporation. The Ag foams were then subjected to a two-step thermal treatment under H_2_. The samples were first heated at 600 °C for 10 min and subsequently annealed at 900 °C. Annealing at 900 °C for 1 h produced THH Cu/Ag. Extending the annealing time at 900 °C to 2 h yielded HOH Cu/Ag. The heating rate was maintained at 30 °C min^–1^, and all samples were quenched to room temperature. Te-free Cu/Ag control catalysts were prepared using a precursor solution containing Cu but no Te. The solution was deposited onto the Ag foam using the same spray procedure, followed by thermal treatment at 600 °C for 10 min and 900 °C for 1 h, and the catalyst was denoted as non-facet-controlled Cu/Ag. A Te-modified non-facet-controlled Cu/Ag catalyst was prepared using the same Cu–Te precursor solution. After spray deposition, the coated Ag foam was heated at 600 °C for 10 min and subsequently annealed at 700 °C for 3 h.

### 2.3 Characterization

The surface morphology and microstructure of the Ag foam and Cu/Ag electrodes were examined by scanning electron microscopy (SEM; SU8600). Elemental distributions were analyzed by energy-dispersive X-ray spectroscopy (EDS) coupled with the SEM. The crystalline structures were characterized by X-ray diffraction (XRD; D8 ADVANCE, Bruker) using Cu Kα radiation (λ = 1.5406 Å) at 40 kV and 40 mA. The composition of the catalytically active region was determined by inductively coupled plasma optical emission spectroscopy (ICP-OES; Optima 8300, PerkinElmer). The coated 1.0 × 1.0 cm^2^ active region was excised from the electrode and digested in 5 mL of fuming HNO_3_ at 120 °C for 0.5 h in a hydrothermal reactor. The resulting solution was diluted to an appropriate concentration prior to ICP-OES analysis.

### 2.4 Electrochemical Measurements

Flow-cell CO_2_ electroreduction measurements were performed using a CHI 760E electrochemical workstation in a three-electrode configuration. Ag foam or Cu/Ag foam was used as the working electrode, IrO_2_-coated Ti felt as the counter electrode, and saturated Ag/AgCl as the reference electrode. The cathodic and anodic compartments were separated by a Nafion 212 membrane. A 0.1 M KHCO_3_ solution was circulated through both compartments at 4 mL min^–1^, while CO_2_ was supplied to the cathode at 25 sccm. The geometric area of the working electrode was 1.0 cm^2^. Chronoamperometric measurements were conducted at potentials from −1.0 to −1.4 V versus the reversible hydrogen electrode (RHE), with each potential maintained for 5 min. The measured potentials were converted to the RHE scale according to *E*_RHE_ = *E*_Ag/AgCl_ + 0.197 + 0.0591 × pH, where the electrolyte pH was measured under the experimental conditions. Unless otherwise specified, all potentials reported in this work are referenced to the RHE and are presented without iR correction. Gas-phase products were quantified online using an Agilent 7890B gas chromatograph equipped with flame ionization and thermal conductivity detectors. Liquid products were analyzed by ^1^H nuclear magnetic resonance spectroscopy using a Bruker Avance III HD 400 MHz spectrometer, with dimethyl sulfoxide as the internal standard. The product FEs were calculated based on the quantified amounts of individual products and the total charge passed during electrolysis. All measurements were independently repeated three times, and the results are presented as the mean ± standard deviation. The long-term electrochemical stability of the catalyst was evaluated by continuous electrolysis at −1.3 V. The first product analysis was performed after 15 min of electrolysis and was taken as the initial sampling point (*t* = 0 h), followed by subsequent analyses at 12 h intervals up to 48 h relative to this initial measurement. The current density was continuously recorded throughout the test, while gas- and liquid-phase products were periodically analyzed to determine the corresponding product selectivity.

The electrochemically active surface area (ECSA) was estimated from the electrochemical double-layer capacitance. Cyclic voltammograms were recorded in a non-Faradaic potential window from −0.15 to −0.05 V at scan rates of 20, 40, 60, 80, 100, and 120 mV s^–1^. The capacitive current difference was plotted against scan rate to obtain the double-layer capacitance, and the ECSA was estimated according to ECSA = *C*_dl_/*C*_s_ using the same specific capacitance value for all samples *C*_s_ = 40 μF cm^–2^. The partial current density of a product or product group was calculated as *j*_partial_ = |*j*_total_| × FE/100. The total C_2_ partial current density included ethylene, ethane, ethanol, and acetate.

### 2.5 In Situ Spectroscopic Measurements

In situ Raman spectra were collected using a Renishaw inVia-Reflex spectrometer with a 532 nm laser in CO_2_-saturated 0.5 M KHCO_3_ electrolyte. Measurements were performed in an H-cell using the Cu/Ag foam electrode as the working electrode, a graphite rod as the counter electrode, and an Ag/AgCl electrode as the reference electrode. Before testing, the electrolyte was purged with CO_2_ for 30 min, and CO_2_ flow was maintained throughout the measurements. Spectra were recorded over 200–2500 cm^–1^ with an acquisition time of 20 s at each applied potential. In situ external-reflection Fourier-transform infrared spectroscopy (FTIR) spectra were collected using a Nicolet iS50 FTIR spectrometer equipped with an MCT detector and a homemade external-reflection accessory. The Cu/Ag foam was used directly as the working electrode, with a Pt wire and an Ag/AgCl electrode serving as the counter and reference electrodes, respectively. A background spectrum was collected at open-circuit potential in CO_2_-saturated 0.5 M KHCO_3_ electrolyte. Potential-dependent spectra were then recorded from −1.0 to −1.4 V in −0.1 V increments, with each potential held for 180 s.

## 3 Results and Discussion

### 3.1 Structural Characterization of High-Index-Faceted Cu/Ag Tandem Catalysts

The overall structure of the Cu/Ag tandem catalysts was first examined at the electrode scale. SEM images show that the pristine Ag foam exhibits an interconnected three-dimensional porous framework with abundant macropores (Figure 2a). Following deposition and thermal treatment of the Cu precursor, Cu nanoparticles were distributed throughout the Ag framework while the interconnected porous architecture of the foam was largely retained (Figures 2b and S1). EDS mapping shows a continuous distribution of Ag across the framework, together with broadly dispersed Cu signals over the Ag surface (Figures 2c,d and S2). The XRD pattern displays diffraction peaks assigned to face-centered cubic Ag and Cu, with no additional crystalline phases detected (Figure 2e). These results collectively confirm the incorporation of crystalline Cu into the porous Ag foam.

**Figure 2:**
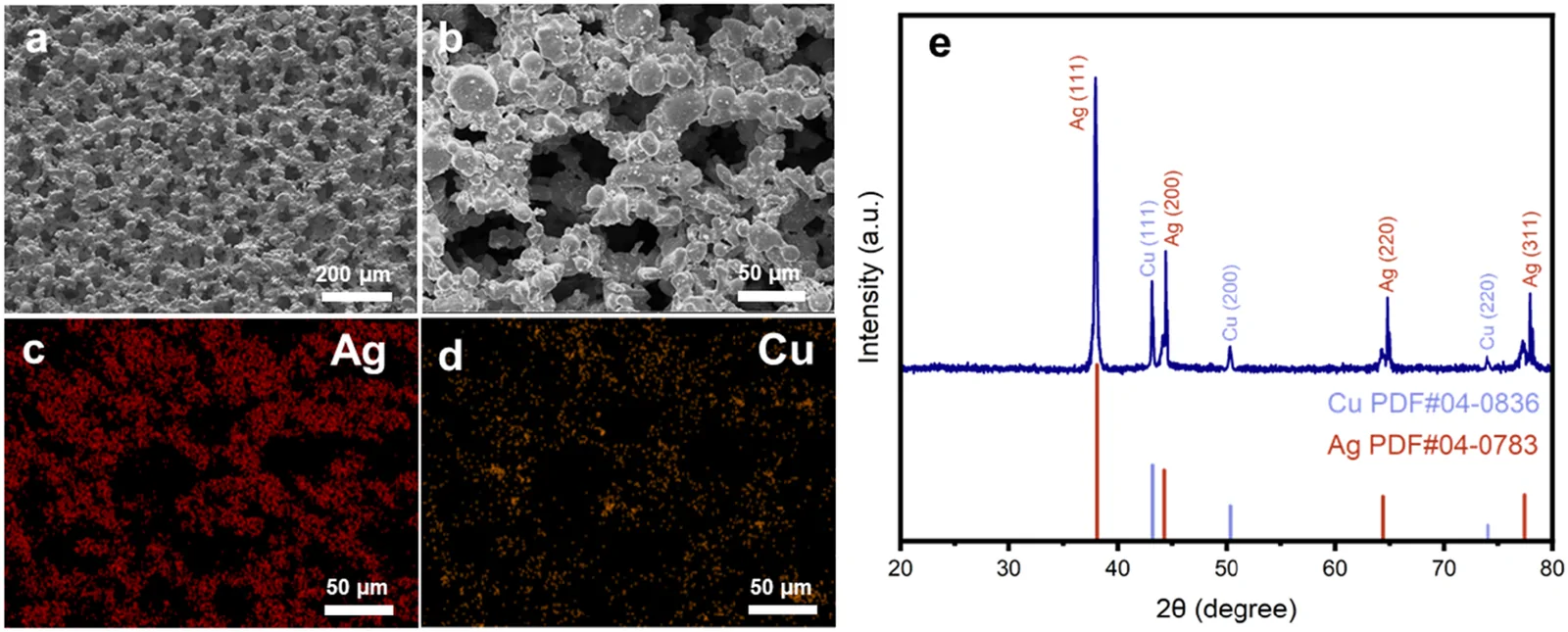
Structural characterization of the Cu/Ag tandem catalyst. SEM image of (a) the pristine Ag foam and (b) the Cu-loaded Ag foam after precursor deposition and thermal treatment. (c,d) Corresponding EDS elemental maps of Ag and Cu, respectively, showing the distribution of Cu over the Ag framework. (e) XRD pattern of the Cu/Ag catalyst, together with the standard diffraction patterns of Cu (PDF No. 04-0836) and Ag (PDF No. 04-0783).

The morphology of the Cu nanoparticles was further examined at higher magnification. Two distinct polyhedral structures with particle sizes ranging from approximately 100 to 500 nm were observed. Well-defined THH Cu nanoparticles are shown in Figure 3a,b, whereas HOH Cu nanoparticles with more densely faceted surfaces are shown in Figure 3c,d. As previously reported, trace amounts of Te modify the Cu surface and stabilize specific high-index facets by regulating the surface energy.^38^ Based on the crystallographic assignments established in the reported protocol, the THH and HOH morphologies have been associated with predominantly exposed {*hk*0} and {*hkl*} facets, respectively.^37^ In this work, the catalysts are therefore denoted as THH Cu/Ag and HOH Cu/Ag according to their experimentally observed morphologies. ICP-OES analysis was further performed to quantify the relative Cu and Te contents. After normalization to the total Cu and Te contents, THH Cu/Ag retained a relatively higher Te content of 0.68%, with Cu accounting for 99.32%. In comparison, the Te content in HOH Cu/Ag decreased to 0.24%, while the Cu content increased to 99.76%. This difference is consistent with the progressive loss of Te with prolonged high-temperature annealing during the formation of THH and HOH Cu nanoparticles.

**Figure 3:**
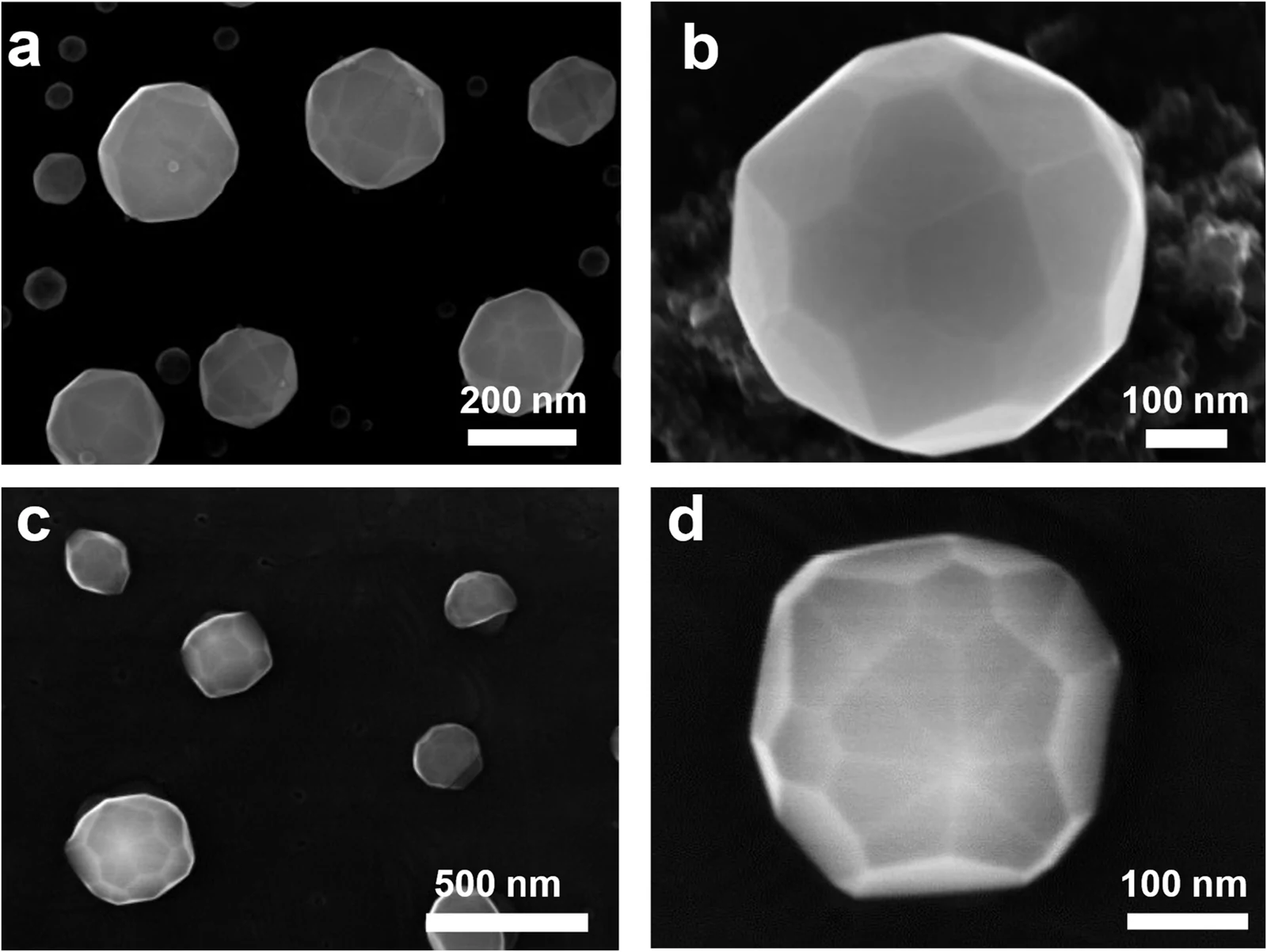
Morphological characterization of high-index-faceted Cu nanoparticles in the Cu/Ag tandem catalysts. (a,b) Low- and high-magnification SEM images of THH Cu nanoparticles. (c,d) Low- and high-magnification SEM images of HOH Cu nanoparticles.

### 3.2 Morphology Dependent CO_2_ Electroreduction Performance

The electrochemical performance of pristine Ag foam, non-facet-controlled Cu/Ag, THH Cu/Ag, and HOH Cu/Ag was systematically evaluated. All three Cu/Ag catalysts contained comparable Cu contents of approximately 40 wt % within the catalytically active region, thereby minimizing the influence of differences in Cu composition on product selectivity. The overall electrochemical activity was first compared using the potential-dependent current densities shown in Figure S3. All Cu-containing catalysts exhibited higher current densities than pristine Ag over the investigated potential range, indicating that the incorporation of Cu promoted the overall cathodic reaction. The product selectivity of the four catalysts was then evaluated from −1.0 to −1.4 V. As shown in Figure 4, pristine Ag foam predominantly produced CO, with a FE of approximately 70–80% throughout the investigated potential range. These results confirm that Ag primarily serves as a CO-generating component and has limited capability for C–C coupling under the present conditions.^39,40^ The introduction of Cu markedly changed the product distribution, leading to the formation of substantial amounts of C_2_^+^ products, mainly ethylene, together with smaller amounts of ethanol, acetate, and other minor products. For all Cu/Ag catalysts, the total C_2_^+^ FE increased as the potential shifted from −1.0 to −1.3 V, accompanied by a gradual decrease in CO selectivity, consistent with the enhanced conversion of Ag-derived CO on adjacent Cu surfaces. At −1.4 V, however, the C_2_^+^ selectivity decreased while hydrogen evolution increased markedly, indicating stronger competition from the hydrogen evolution reaction. The ethylene selectivity reached a maximum at −1.3 V for all three Cu/Ag catalysts, following the order HOH Cu/Ag > THH Cu/Ag > non-facet-controlled Cu/Ag, with FEs of 60.37%, 53.34%, and 40.11%, respectively (Figure 4b–d). Notably, the differences in total current density among the three Cu/Ag catalysts were relatively modest, whereas the C_2_ partial current densities followed the order of HOH Cu/Ag > THH Cu/Ag > non-facet-controlled Cu/Ag, consistent with the trend in ethylene selectivity (Figure S4). These results indicate the important role of high-index-faceted Cu nanoparticle morphology in regulating reaction pathways and product distribution during tandem CO_2_ electroreduction. It should be noted that differences in catalyst morphologies may influence not only the intrinsic adsorption and coupling energetics of reaction intermediates, but also the local reaction environment.^28,41–43^ Therefore, these effects should be regarded as coupled consequences of catalyst morphology under practical operating conditions.^44^

**Figure 4:**
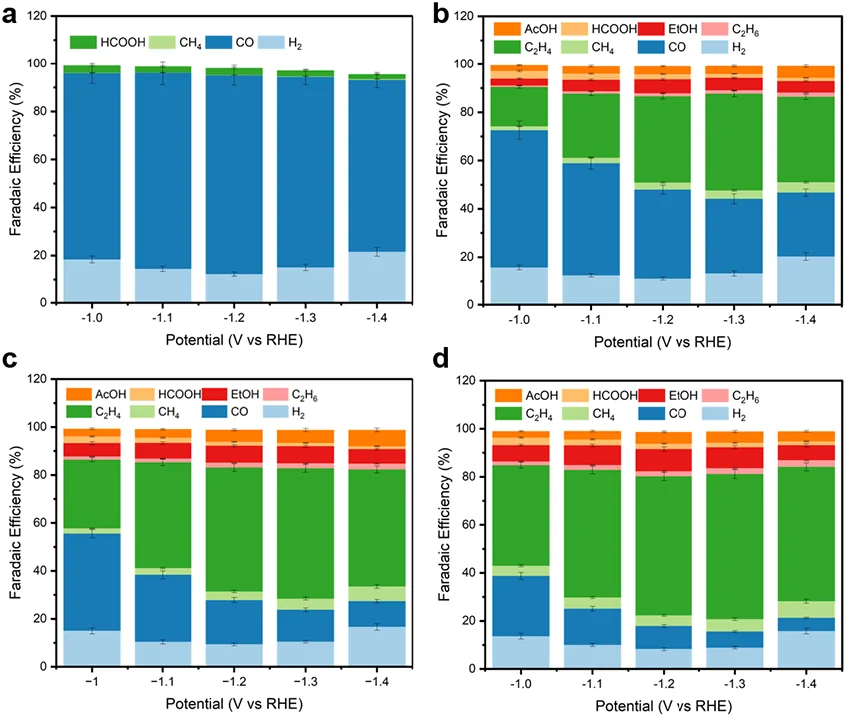
Potential-dependent product distributions for CO_2_ electroreduction of various catalysts. (a) Pristine Ag foam, (b) non-facet-controlled Cu/Ag, (c) THH Cu/Ag, and (d) HOH Cu/Ag. Error bars represent the standard deviations from three independent measurements.

The formation of distinct Cu nanoparticle morphologies may also be accompanied by changes in other catalyst properties, such as the electrochemically accessible surface area and residual Te content, which could influence the observed selectivity. We therefore examined these factors to clarify their possible contributions to the morphology-dependent catalytic behavior. ECSAs estimated from double-layer-capacitance measurements were 10.90, 10.83, and 11.05 cm^2^ for non-facet-controlled Cu/Ag, THH Cu/Ag, and HOH Cu/Ag, respectively (Figure S5). The maximum difference was only 0.22 cm^2^, or approximately 2.0% of the average ECSA, indicating essentially comparable electrochemically accessible surface areas. Moreover, a Te-modified Cu/Ag catalyst without a well-defined high-index-faceted morphology was examined as a control. This control was annealed at 700 °C for 3 h to retain a non-facet-controlled Cu morphology while allowing substantial thermal treatment of the Cu/Te precursor. Despite having a Te content of 0.45%, comparable in magnitude to those of THH Cu/Ag and HOH Cu/Ag, this catalyst exhibited an ethylene FE of only 27.64%, which was even lower than that of Te-free non-facet-controlled Cu/Ag (Figure S6). This result is consistent with the reported role of Te primarily as a structure-directing species during the formation of high-index-faceted Cu nanoparticles.^38^ Although possible local Te-mediated effects cannot be completely excluded, the substantially lower ethylene selectivity of this control indicates that residual Te alone is insufficient to explain the enhanced performance of the THH and HOH Cu/Ag catalysts. These results indicate that the selectivity trend is governed primarily by the exposed Cu surface structure rather than differences in accessible surface area or residual Te content.

### 3.3 In Situ Spectroscopic Analysis

To gain insight into the origin of the enhanced ethylene formation, potential-dependent in situ Raman and external-reflection FTIR measurements were performed on HOH Cu/Ag, which exhibited the highest ethylene selectivity among the investigated catalysts, under CO_2_ reduction conditions. As shown in Figure 5a, the Raman spectrum collected at open-circuit potential exhibits bands attributable to surface Cu–O and Cu_2_O species at 218, 298, 400, 528, and 620 cm^–1^.^44^ These oxide-related features gradually weaken after applying cathodic potentials, indicating the reduction of surface Cu oxide species and the formation of a predominantly metallic Cu surface under working conditions.

**Figure 5:**
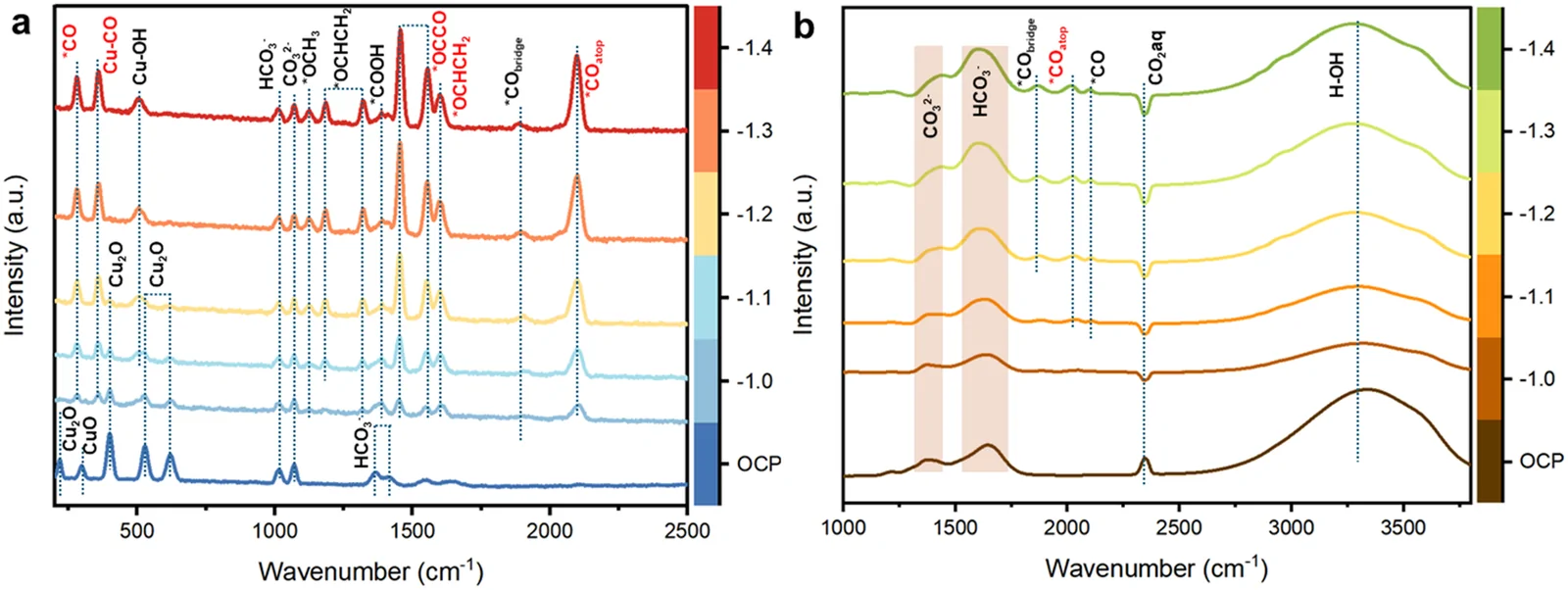
Potential-dependent in situ spectroscopic characterization of HOH Cu/Ag during CO_2_ electroreduction. (a) In situ Raman spectra collected from the open-circuit potential (OCP) to −1.4 V. (b) In situ FTIR spectra collected over the same potential range.

Upon polarization, bands associated with adsorbed CO species become increasingly pronounced. The low-wavenumber features assigned to Cu–CO vibrations at 358 cm^–1^, together with the bands attributed to bridge-bonded CO at 1910 cm^–1^ and atop-bonded CO at 2100 cm^–1^,^45–52^ increase progressively as the potential becomes more negative. This trend indicates continuous generation and accumulation of CO-derived intermediates on the HOH Cu/Ag surface. The simultaneous observation of carbonate species at 1070 cm^–1^ and bicarbonate species at 1010 cm^–1^ reflects the interfacial CO_2_-electrolyte equilibrium under reaction conditions.^45–52^ More importantly, several potential-dependent bands emerging at increasingly negative potentials can be tentatively assigned to hydrogenated and coupled CO intermediates, including *COOH at 1390 cm^–1^, *OCCO at 1450 and 1550 cm^–1^, and *OCHCH_2_ species at 1182, 1318, and 1595 cm^–1^. In particular, the growth of the *OCCO-related band at 1450 and 1550 cm^–1^ provides spectroscopic evidence for enhanced coupling between adsorbed CO intermediates, which represents a critical step toward C_2_^+^ product formation. The appearance of hydrogenated C_2_ intermediates at approximately 1182, 1318, and 1595 cm^–1^ further suggests that the coupled species undergo subsequent proton-electron transfer toward ethylene and other multicarbon products.^45^

The in situ FTIR spectra show a consistent potential-dependent evolution (Figure 5b). Bands associated with bicarbonate and carbonate species are observed in the 1300–1700 cm^–1^ region, while signals assigned to bridge-bonded CO at 1885 cm^–1^ and atop-bonded CO appear at 2018 cm^–1^.^49^ These CO-related features become more evident under increasingly negative potentials, confirming the accumulation of surface-bound CO intermediates. Meanwhile, the feature near 2343 cm^–1^ is attributed to dissolved CO_2_, and the broad band in the O–H stretching (3200–3600 cm^–1^) region reflects changes in the interfacial water and hydrogen-bonding environment during electrolysis. Taken together, the in situ Raman and FTIR results reveal the potential-dependent conversion of the HOH Cu/Ag surface from an initially oxidized state to an active metallic Cu surface, followed by the accumulation and coupling of CO-derived intermediates. The simultaneous observation of abundant adsorbed CO and C–C coupling intermediates is consistent with the high ethylene FE of HOH Cu/Ag.

### 3.4 Potential-Dependent Stability and Surface Reconstruction

The structural stability of high-index-faceted Cu catalysts is an important consideration during CO_2_ electroreduction because Cu surfaces may undergo potential-dependent reconstruction under cathodic operating conditions, potentially altering the exposed surface structures and product selectivity over prolonged electrolysis.^53–58^ The long-term stability of HOH Cu/Ag was therefore evaluated by chronoamperometric electrolysis at −1.3 V for 48 h. As shown in Figure 6a, the catalyst maintained a relatively high current density throughout the test, although a gradual decrease from approximately 150 to 125 mA cm^–2^ was observed. In comparison, the ethylene FE remained relatively stable, showing only a slight decrease and reaching 54.7% after 48 h. The product distribution measured after the stability test was also broadly comparable to that obtained initially, indicating that the ethylene–selective reaction pathway was largely maintained (Figure 6c). A post-reaction SEM image further shows that the Cu nanoparticles largely retained their characteristic HOH morphology after operation at −1.3 V (Figure 6b). These results indicate that HOH Cu/Ag can maintain relatively stable ethylene selectivity and substantial structural integrity under the optimal operating conditions. The stability was further examined at the more negative potential of −1.4 V. After 12 h of electrolysis, the catalyst already exhibited a pronounced decline in catalytic performance, together with substantial surface roughening and partial loss of the initially well-defined HOH morphology (Figure S7). Upon extending the electrolysis to 48 h, the HOH structure was largely destroyed, accompanied by a further decrease in ethylene selectivity (Figure S8). These observations suggest that stronger cathodic polarization accelerates surface reconstruction of the catalyst and that the progressive loss of the characteristic HOH morphology is associated with declining ethylene selectivity.

**Figure 6:**
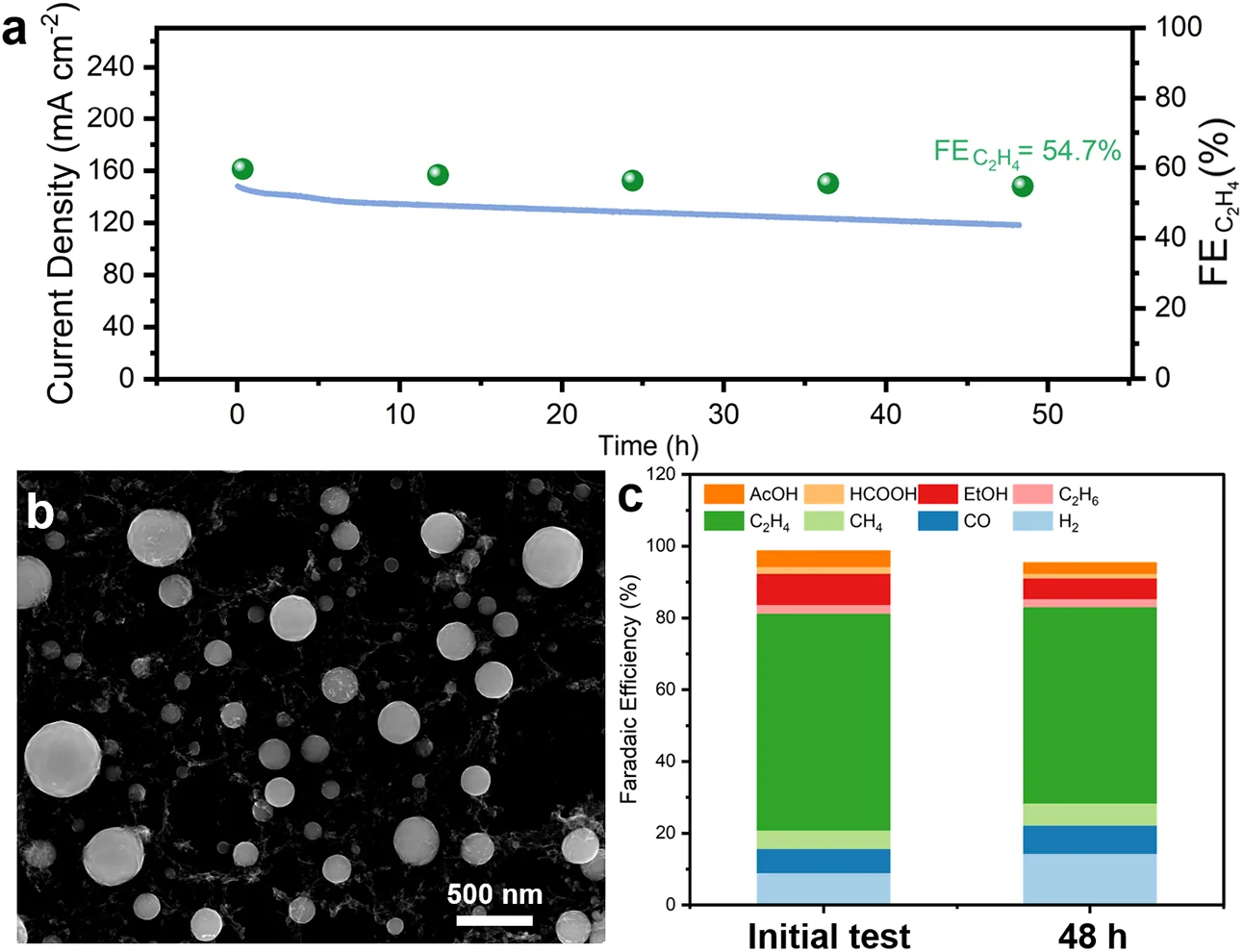
Structural stability of HOH Cu/Ag during CO_2_ electroreduction. (a) Current density and ethylene FE during the 48 h stability test. (b) SEM image of HOH-shaped Cu particles after 48 h of electrolysis. (c) Product distributions measured at the initial sampling point and 48 h after the initial measurement.

## 4 Conclusion

In this work, two types of Cu nanoparticles with distinct high-index-faceted morphologies were loaded onto Ag foam to construct Cu/Ag tandem catalysts for CO_2_ electroreduction. The ethylene selectivity followed the order HOH Cu/Ag > THH Cu/Ag > non-facet-controlled Cu/Ag, with HOH Cu/Ag achieving a FE of 60.37% at −1.3 V. In situ spectroscopy further revealed abundant adsorbed CO species and possible CO-derived coupling intermediates on HOH Cu/Ag within the potential range associated with pronounced ethylene production. More importantly, HOH Cu/Ag largely retained its characteristic morphology and ethylene selectivity during prolonged electrolysis at −1.3 V, whereas operation at −1.4 V induced surface roughening and pronounced performance decay. Overall, this work demonstrates the catalytic advantage associated with high-index-faceted Cu morphologies in Ag–Cu tandem systems, while also showing that maintaining such an advantage requires preservation of the characteristic morphology under operating conditions. These findings suggest that future shape-controlled catalysts should be designed by jointly considering morphology, reaction potential, and tandem architecture to identify and broaden the operating window in which catalytically favorable surface structures can be maintained.

## Supplementary Material



## Notes

Additional materials characterization, including SEM images, EDS mappings, and electrochemical tests (PDF)

## References

[1] Shafaat H. S., Yang J. Y. (2021). Uniting Biological and Chemical Strategies for Selective
CO_2_ Reduction. Nat. Catal..

[2] Jin S., Hao Z., Zhang K., Yan Z., Chen J. (2021). Advances and Challenges
for the Electrochemical Reduction of CO_2_ to CO: From Fundamentals
to Industrialization. Angew. Chem., Int. Ed..

[3] Zhu P., Wang H. (2021). High-Purity and High-Concentration Liquid Fuels through
CO_2_ Electroreduction. Nat. Catal..

[4] Nitopi S., Bertheussen E., Scott S. B., Liu X., Engstfeld A. K., Horch S., Seger B., Stephens I. E. L., Chan K., Hahn C. (2019). Progress and Perspectives of Electrochemical CO_2_ Reduction on Copper in Aqueous Electrolyte. Chem. Rev..

[5] Zheng Y., Vasileff A., Zhou X., Jiao Y., Jaroniec M., Qiao S.-Z. (2019). Understanding the
Roadmap for Electrochemical Reduction
of CO_2_ to Multi-Carbon Oxygenates and Hydrocarbons on Copper-Based
Catalysts. J. Am. Chem. Soc..

[6] Cheng D., Zhao Z.-J., Zhang G., Yang P., Li L., Gao H., Liu S., Chang X., Chen S., Wang T. (2021). The Nature of Active Sites for Carbon Dioxide Electroreduction over
Oxide-Derived Copper Catalysts. Nat. Commun..

[7] Rihm S. D., Kovalev M. K., Lapkin A. A., Ager J. W., Kraft M. (2023). On the Role
of C_4_ and C_5_ Products in Electrochemical CO_2_ Reduction via Copper-Based Catalysts. Energy Environ. Sci..

[8] Zhang H., Gao J., Raciti D., Hall A. S. (2023). Promoting Cu-Catalysed CO_2_ Electroreduction
to Multicarbon Products by Tuning the Activity
of H_2_O. Nat. Catal..

[9] Chen J., Wang D., Yang X., Cui W., Sang X., Zhao Z., Wang L., Li Z., Yang B., Lei L. (2023). Accelerated Transfer
and Spillover of Carbon Monoxide
through Tandem Catalysis for Kinetics-Boosted Ethylene Electrosynthesis. Angew. Chem., Int. Ed..

[10] Bian L., Bai Y., Chen J.-Y., Guo H.-K., Liu S., Tian H., Tian N., Wang Z.-L. (2025). Hierarchical Tandem Catalysis Promotes
CO Spillover and Trapping for Efficient CO_2_ Reduction to
C_2_
^+^ Products. ACS Nano.

[11] Pan H., Wu J., Lu X., Li Y., Zhang Q., Ling J., Wang Y., Min B. (2025). Nanoporous Bimetallic
Ag–Cu
Tandem Electrocatalysts for Electrochemical CO_2_ Reduction
to Multicarbon Products via Asymmetric C–C Coupling. ACS Appl. Energy Mater..

[12] Chen C., Li Y., Yu S., Louisia S., Jin J., Li M., Ross M. B., Yang P. (2020). Cu–Ag Tandem Catalysts for
High-Rate CO_2_ Electrolysis toward Multicarbons. Joule.

[13] Morales-Guio C. G., Cave E. R., Nitopi S. A., Feaster J. T., Wang L., Kuhl K. P., Jackson A., Johnson N. C., Abram D. N., Hatsukade T. (2018). Improved CO_2_ Reduction Activity
toward C_2_
^+^ Alcohols on a Tandem Gold-on-Copper
Electrocatalyst. Nat. Catal..

[14] Ashworth C. (2018). Top Tandem
Team. Nat. Rev. Mater..

[15] Yang R., Duan J., Dong P., Wen Q., Wu M., Liu Y., Liu Y., Li H., Zhai T. (2022). In Situ Halogen-Ion
Leaching Regulates Multiple Sites on Tandem Catalysts for Efficient
CO_2_ Electroreduction to C_2_
^+^ Products. Angew. Chem., Int. Ed..

[16] Du C., Mills J. P., Yohannes A. G., Wei W., Wang L., Lu S., Lian J.-X., Wang M., Guo T., Wang X. (2023). Cascade Electrocatalysis via AgCu Single-Atom Alloy and Ag Nanoparticles
in CO2 Electroreduction toward Multicarbon Products. Nat. Commun..

[17] Zhang T., Bui J. C., Li Z., Bell A. T., Weber A. Z., Wu J. (2022). Highly Selective and
Productive Reduction of Carbon Dioxide to Multicarbon
Products via In Situ CO Management Using Segmented Tandem Electrodes. Nat. Catal..

[18] Sassenburg M., Iglesias van Montfort H. P., Kolobov N., Smith W. A., Burdyny T. (2025). Bulk Layering Effects
of Ag and Cu for Tandem CO_2_ Electrolysis. ChemSusChem.

[19] Zhang J., Huang J., Zhang S., Cui Y., Kong C., Peng Z., Jiang H., Deng S., Chen Z., Yang C. (2026). Confinement-Driven CO Spillover in CuAg@MSN Tandem
Catalysts Boosts C2 Selectivity toward Electrocatalytic CO_2_ Reduction. Adv. Sci..

[20] Lum Y., Ager J. W. (2019). Evidence for Product-Specific
Active Sites on Oxide-Derived
Cu Catalysts for Electrochemical CO_2_ Reduction. Nat. Catal..

[21] Amirbeigiarab R., Tian J., Herzog A., Qiu C., Bergmann A., Roldan Cuenya B., Magnussen O. M. (2023). Atomic-Scale
Surface Restructuring
of Copper Electrodes under CO_2_ Electroreduction Conditions. Nat. Catal..

[22] Gao W., Xu Y., Fu L., Chang X., Xu B. (2023). Experimental Evidence
of Distinct Sites for CO_2_-to-CO and CO Conversion on Cu
in the Electrochemical CO_2_ Reduction Reaction. Nat. Catal..

[23] Wu Q., Du R., Wang P., Waterhouse G. I. N., Li J., Qiu Y., Yan K., Zhao Y., Zhao W.-W., Tsai H.-J. (2023). Nanograin-Boundary-Abundant
Cu_2_O–Cu Nanocubes with High C_2_
^+^ Selectivity and Good Stability during Electrochemical CO_2_ Reduction at a Current Density of 500 mA/cm^2^. ACS Nano.

[24] Qiao Y., Shen S., Mao C., Xiao Y., Lai W., Wang Y., Zhong X., Lu Y., Li J., Ge J. (2025). Interfacial Oxygen Vacancy–Copper
Pair Sites
on Inverse CeO_2_/Cu Catalyst Enable Efficient CO_2_ Electroreduction to Ethanol in Acid. Angew.
Chem., Int. Ed..

[25] Du R., Wu Q., Zhang S., Wang P., Li Z., Qiu Y., Yan K., Waterhouse G. I. N., Wang P., Li J. (2023). Cu–C­(O) Interfaces Deliver Remarkable Selectivity and Stability
for CO_2_ Reduction to C_2_
^+^ Products
at an Industrial Current Density of 500 mA/cm^2^. Small.

[26] Du R., Li T., Wu Q., Wang P., Yang X., Fan Y., Qiu Y., Yan K., Wang P., Zhao Y. (2022). In Situ
Engineering of the Cu^+^/Cu^0^ Interface to Boost
C_2_
^+^ Selectivity in CO_2_ Electroreduction. ACS Appl. Mater. Interfaces.

[27] Zhang Z., Bian L., Tian H., Liu Y., Bando Y., Yamauchi Y., Wang Z.-L. (2022). Tailoring the Surface and Interface
Structures of Copper-Based Catalysts for Electrochemical Reduction
of CO_2_ to Ethylene and Ethanol. Small.

[28] Pan F., Yang Y. (2020). Designing CO_2_ Reduction Electrode Materials by Morphology
and Interface Engineering. Energy Environ. Sci..

[29] Wahab O. J., Kang M., Daviddi E., Walker M., Unwin P. R. (2022). Screening
Surface Structure–Electrochemical Activity Relationships of
Copper Electrodes under CO_2_ Electroreduction Conditions. ACS Catal..

[30] Liu X., Xiao J., Peng H., Hong X., Chan K., Nørskov J. K. (2017). Understanding
Trends in Electrochemical Carbon Dioxide
Reduction Rates. Nat. Commun..

[31] Zhong D., Zhao Z.-J., Zhao Q., Cheng D., Liu B., Zhang G., Deng W., Dong H., Zhang L., Li J. (2021). Coupling of Cu(100) and Cu(110) Facets Promotes Carbon
Dioxide Conversion to Hydrocarbons and Alcohols. Angew. Chem., Int. Ed..

[32] Bagger A., Ju W., Varela A. S., Strasser P., Rossmeisl J. (2019). Electrochemical
CO_2_ Reduction: Classifying Cu Facets. ACS Catal..

[33] Philip M., Woldu A. R., Akbar M. B., Louis H., Cong H. (2021). A Facile Synthesis
of Cu Catalysts with Multiple High-Index Facets for the Suppression
of Competing H_2_ Evolution during Electrocatalytic CO_2_ Reduction. Nanoscale.

[34] Yao K., Li J., Ozden A., Wang H., Sun N., Liu P., Zhong W., Zhou W., Zhou J., Wang X. (2024). In Situ Copper Faceting Enables Efficient CO_2_/CO Electrolysis. Nat. Commun..

[35] Bian L., Zhang Z.-Y., Tian H., Tian N.-N., Ma Z., Wang Z.-L. (2023). Grain Boundary-Abundant
Copper Nanoribbons on Balanced
Gas–Liquid Diffusion Electrodes for Efficient CO_2_ Electroreduction to C_2_H_4_. Chin. J. Catal..

[36] Xie L., Liu Y., Li S., Shen B., Zhu W. (2026). Phase and Facet Engineering
for Nanoscale Catalyst Discovery in Electrochemical CO_2_ Reduction. Nat. Synth..

[37] Xiao C., Lu B.-A., Xue P., Tian N., Zhou Z.-Y., Lin X., Lin W.-F., Sun S.-G. (2020). High-Index-Facet-
and High-Surface-Energy
Nanocrystals of Metals and Metal Oxides as Highly Efficient Catalysts. Joule.

[38] Ye Z., Shen B., Kang D., Huang J., Wang Z., Wahl C. B., Shin D., Huang L., Shen J., Wolverton C. M., Mirkin C. A. (2024). Using Surface Composition and Energy
to Control the Formation of Either Tetrahexahedral or Hexoctahedral
High-Index Facet Nanostructures. J. Am. Chem.
Soc..

[39] Ma M., Trześniewski B. J., Xie J., Smith W. A. (2016). Selective
and Efficient Reduction of Carbon Dioxide to Carbon Monoxide on Oxide-Derived
Nanostructured Silver Electrocatalysts. Angew.
Chem., Int. Ed..

[40] Lu Q., Rosen J., Zhou Y., Hutchings G. S., Kimmel Y. C., Chen J. G., Jiao F. A. (2014). Selective
and Efficient
Electrocatalyst for Carbon Dioxide Reduction. Nat. Commun..

[41] Chen X., Chen C., Wang Y., Pan Z., Chen J., Xu Y., Zhu L., Song T., Li R., Chen L., Lu J. (2024). Interfacial Microenvironment Effects on Electrochemical CO_2_ Reduction. Chem. Eng. J..

[42] Zhang J., Zeng G., Zhu S., Tao H., Pan Y., Lai W., Bao J., Lian C., Su D., Shao M., Huang H. (2023). Steering CO_2_ Electroreduction Pathway toward Ethanol via
Surface-Bounded Hydroxyl Species-Induced Noncovalent Interaction. Proc. Natl. Acad. Sci. U.S.A..

[43] Zhang Z.-Y., Tian H., Jiao H., Wang X., Bian L., Liu Y., Khaorapapong N., Yamauchi Y., Wang Z.-L. (2024). SiO_2_-Assisted
Cu^0^–Cu^+^–NH_2_ Composite
Interfaces for Efficient CO_2_ Electroreduction to C_2_
^+^ Products. J. Mater. Chem.
A.

[44] Wang Y., Lai W., Tao H., Qiao Y., Chen X., Lian C., Ge J., Li J., Huang H. (2025). CO_2_ Electroreduction to
Multicarbon Products over Cu_2_O@Mesoporous SiO_2_ Confined Catalyst: Relevance of the Shell Thickness. Adv. Energy Mater..

[45] Jiang S., D’Amario L., Dau H. (2022). Copper Carbonate Hydroxide as Precursor
of Interfacial CO in CO_2_ Electroreduction. ChemSusChem.

[46] Moradzaman M., Mul G. (2020). Infrared Analysis of Interfacial Phenomena during Electrochemical
Reduction of CO_2_ over Polycrystalline Copper Electrodes. ACS Catal..

[47] Zhan C., Dattila F., Rettenmaier C., Bergmann A., Kühl S., García-Muelas R., López N., Cuenya B. R. (2021). Revealing the CO
Coverage-Driven C–C Coupling Mechanism for Electrochemical
CO_2_ Reduction on Cu_2_O Nanocubes via Operando
Raman Spectroscopy. ACS Catal..

[48] Zhan C., Dattila F., Rettenmaier C., Herzog A., Herran M., Wagner T., Scholten F., Bergmann A., López N., Roldan Cuenya B. (2024). Key Intermediates
and Cu Active Sites for CO_2_ Electroreduction to Ethylene
and Ethanol. Nat. Energy.

[49] Kong S., Lv X., Wang X., Liu Z., Li Z., Jia B., Sun D., Yang C., Liu L., Guan A. (2023). Delocalization
State-Induced Selective Bond Breaking for Efficient Methanol Electrosynthesis
from CO_2_. Nat. Catal..

[50] Moradzaman M., Mul G. (2021). In Situ Raman Study of Potential-Dependent
Surface-Adsorbed Carbonate,
CO, OH, and C Species on Cu Electrodes during Electrochemical Reduction
of CO_2_. ChemElectroChem.

[51] El-Nagar G. A., Yang F., Stojkovikj S., Mebs S., Gupta S., Ahmet I. Y., Dau H., Mayer M. T. (2022). Comparative Spectroscopic
Study Revealing Why the CO_2_ Electroreduction Selectivity
Switches from CO to HCOO^–^ at Cu–Sn- and Cu–In-Based
Catalysts. ACS Catal..

[52] Herzog A., Lopez Luna M., Jeon H. S., Rettenmaier C., Grosse P., Bergmann A., Roldan Cuenya B. (2024). Operando Raman
Spectroscopy Uncovers Hydroxide and CO Species Enhance Ethanol Selectivity
during Pulsed CO_2_ Electroreduction. Nat. Commun..

[53] Kim I., Lee G.-B., Kim S., Jung H. D., Kim J.-Y., Lee T., Choi H., Jo J., Kang G., Oh S.-H. (2025). Unveiling the Reconstruction
of Copper Bimetallic Catalysts during
CO_2_ Electroreduction. Nat. Catal..

[54] Vavra J., Ramona G. P. L., Dattila F., Kormányos A., Priamushko T., Albertini P. P., Loiudice A., Cherevko S., López N., Buonsanti R. (2024). Solution-Based Cu^+^ Transient
Species Mediate the Reconstruction of Copper Electrocatalysts for
CO_2_ Reduction. Nat. Catal..

[55] Yang Y., Feijóo J., Figueras-Valls M., Chen C., Shi C., Fonseca
Guzman M. V., Murhabazi Maombi Y., Liu S., Jain P., Briega-Martos V. (2025). Operando Probing Dynamic Migration of Copper
Carbonyl during Electrocatalytic CO_2_ Reduction. Nat. Catal..

[56] Yang Y., Louisia S., Yu S., Jin J., Roh I., Chen C., Fonseca Guzman M. V., Feijóo J., Chen P.-C., Wang H. (2023). Operando
Studies Reveal
Active Cu Nanograins for CO_2_ Electroreduction. Nature.

[57] Zhang Z., Gee W., Sautet P., Alexandrova A. N. (2024). H and CO Co-Induced Roughening of
Cu Surface in CO_2_ Electroreduction Conditions. J. Am. Chem. Soc..

[58] Xie Z., Wang Q., Yang H., Feng J., Chen J., Song S., Meng C., Wang K., Tong Y. (2024). Surface Facets
Reconstruction in Copper-Based Materials for Enhanced Electrochemical
CO_2_ Reduction. Small.

