# Supplementary material for: High-Index-Faceted Cu Nanoparticles in Ag–Cu Tandem Catalysts for Selective CO2 Electroreduction
Source: ACS Nanosci Au. 2026 Sep 7;6(5):1029–36. doi: 10.1021/acsnanoscienceau.6c00129 (PMC13692753; doi:10.1021/acsnanoscienceau.6c00129)
Supplement: Supplementary file 1 [file ng6c00129_si_001.pdf]

High-Index-Faceted Cu Nanoparticles in Ag-Cu Tandem Catalysts for Selective CO_2 Electroreduction

Guangtao Wang[1,2], Simin Li[1], Chao Yu[3,4], Yang Zhou[5], Wenlei Zhu[2,], Bo Shen [1,*]*

1. Department of Materials Science and Engineering, City University of Hong Kong, Kowloon, Hong Kong SAR, China

2. State Key Laboratory of Water Pollution Control and Green Resource Recycling, State Key Laboratory of Analytical Chemistry for Life Science, Frontiers Science Center for Critical Earth Material Cycling, School of Environment, School of Chemistry and Chemical Engineering, Nanjing University, Nanjing, 210023, China

3. School of Environmental and Chemical Engineering, Jiangsu University of Science and Technology, Zhenjiang, 212100, China

4. Research Institute of Tsinghua University in Shenzhen, Shenzhen, 518057, China

5. State Key Laboratory of Flexible Electronics (LoFE), Institute of Advanced Materials (IAM), Nanjing University of Posts and Telecommunications (NJUPT), Nanjing 210023, China

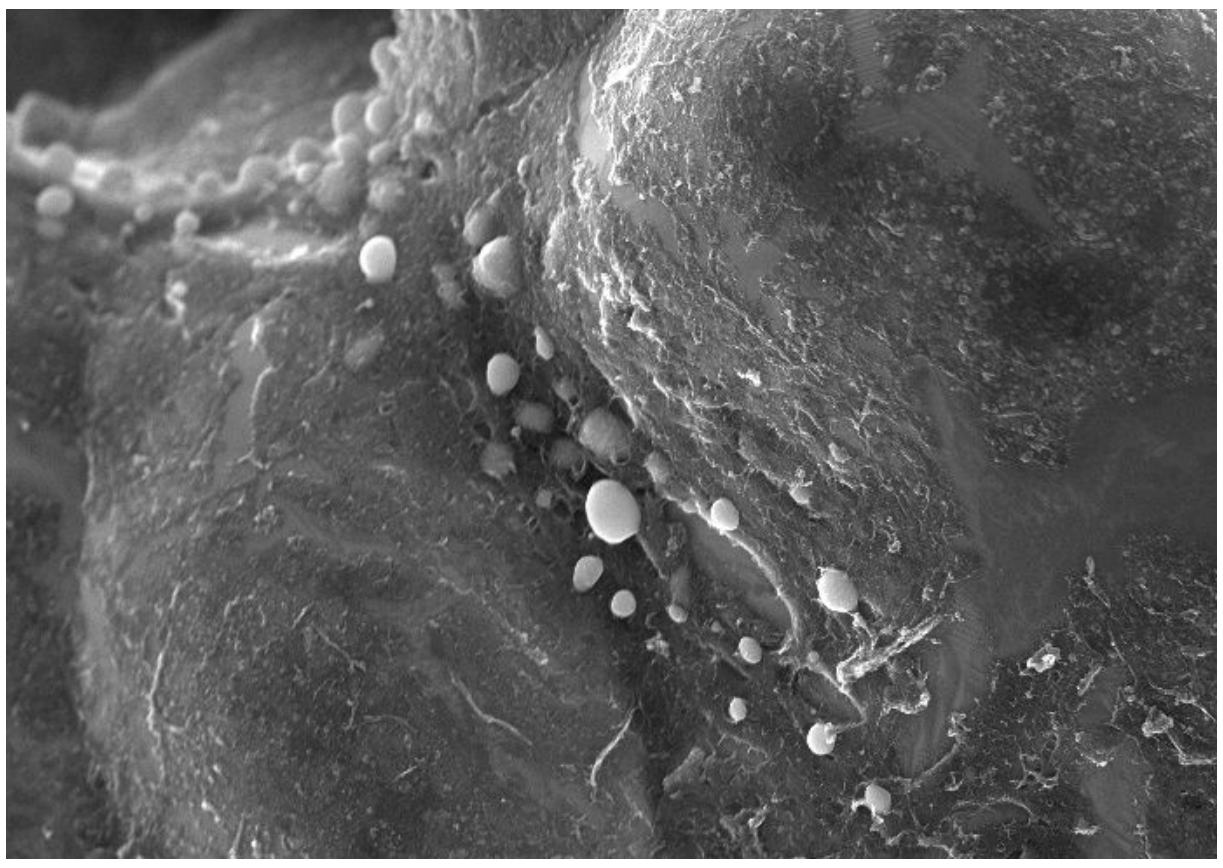

Figure S1. High-magnification SEM image of the Cu-loaded Ag foam, showing Cu-containing particles distributed on the surface of the Ag framework.

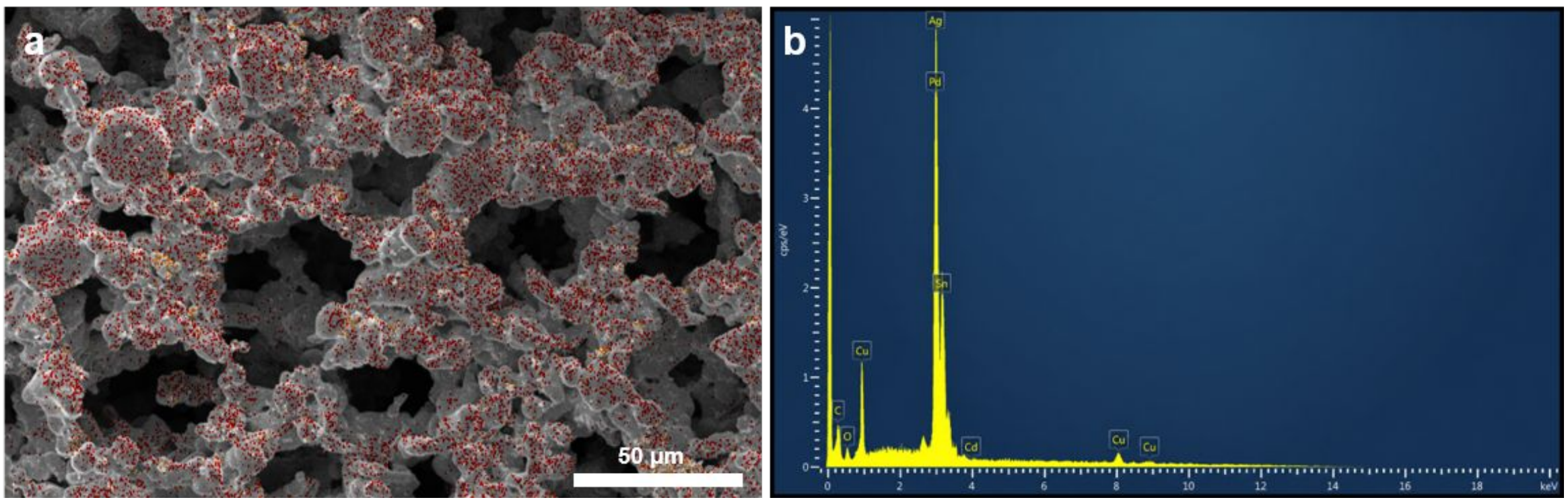

Figure S2. EDS characterization of the Ag–Cu tandem catalyst. Left: overlaid SEM and EDS elemental maps showing the spatial distributions of Ag and Cu across the porous framework. Right: corresponding EDS spectrum confirming the presence of Ag and Cu in the catalyst.

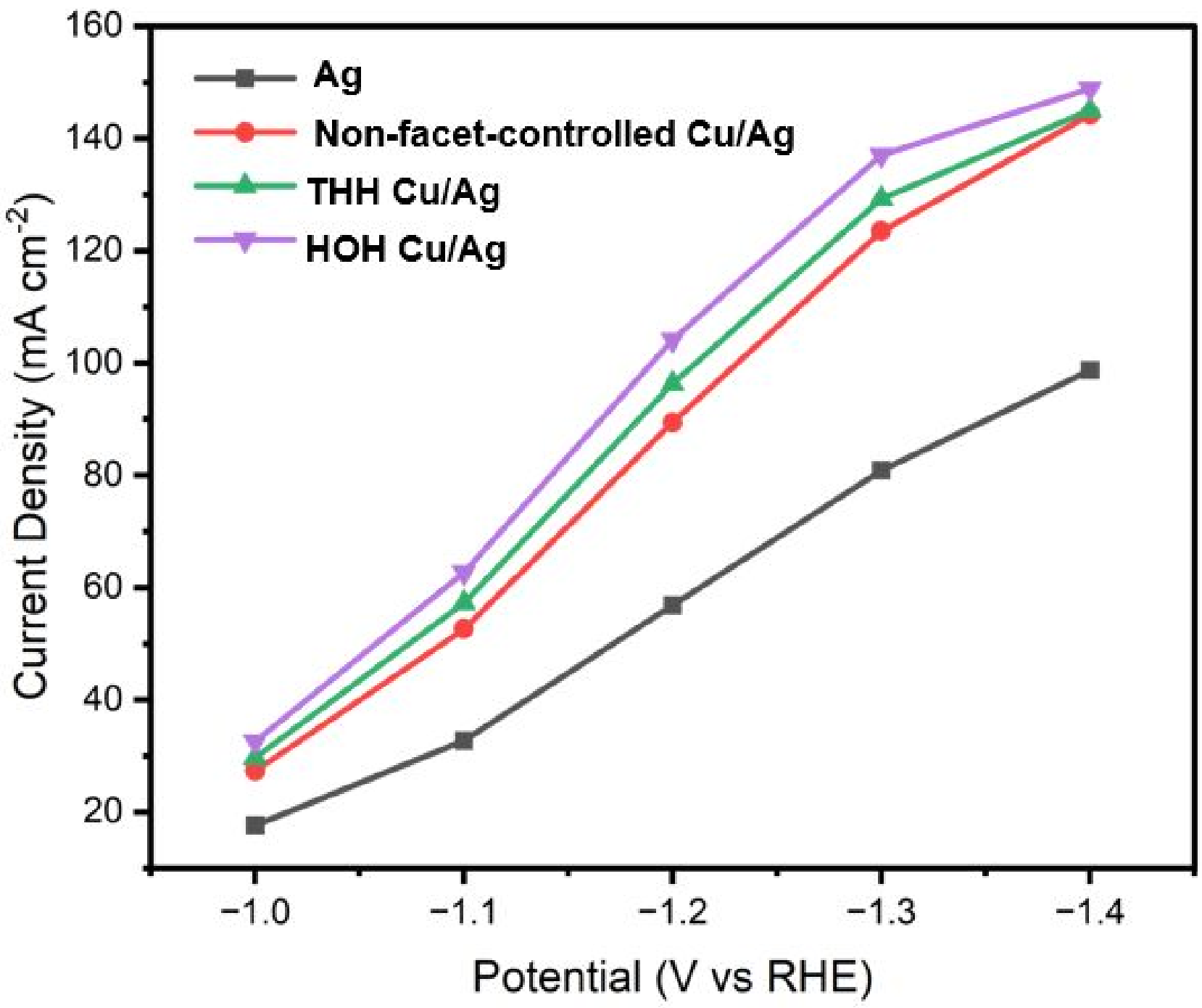

Figure S3. Potential-dependent current densities of Ag, non-facet-controlled Cu/Ag, THH Cu/Ag, and HOH Cu/Ag during CO_2 electroreduction. The current density increases with increasingly negative applied potential for all catalysts.

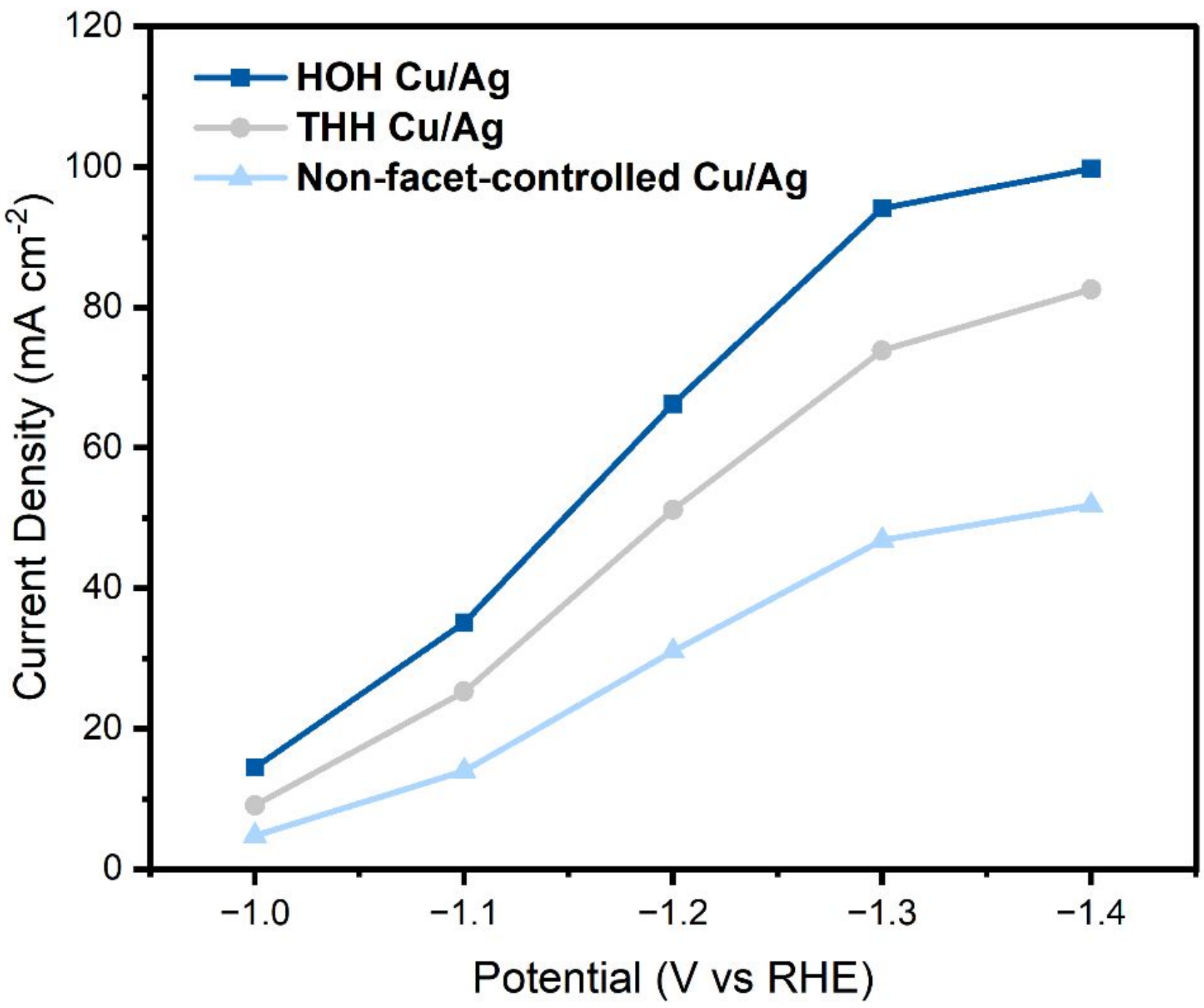

Figure S4. C_2 product partial current densities of non-facet-controlled Cu/Ag, THH Cu/Ag, and HOH Cu/Ag during CO_2 electroreduction.

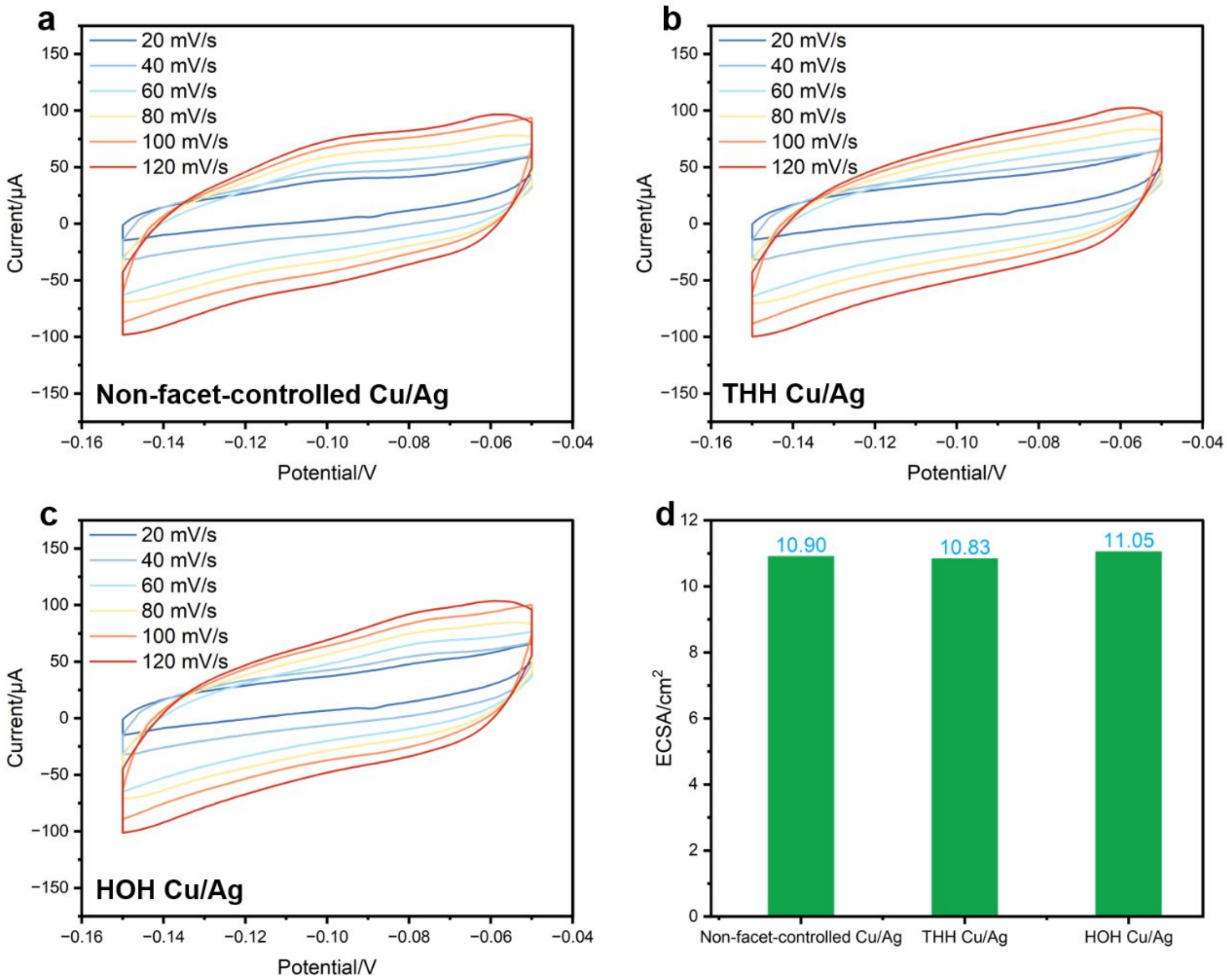

Figure S5. Cyclic voltammetry curves of (a) non-facet-controlled Cu/Ag, (b) THH Cu/Ag, and (c) HOH Cu/Ag recorded at scan rates of 20, 40, 60, 80, 100, and 120 mV s^{-1} in the non-Faradaic potential region. (d) Calculated ECSA values of non-facet-controlled Cu/Ag, THH Cu/Ag, and HOH Cu/Ag.

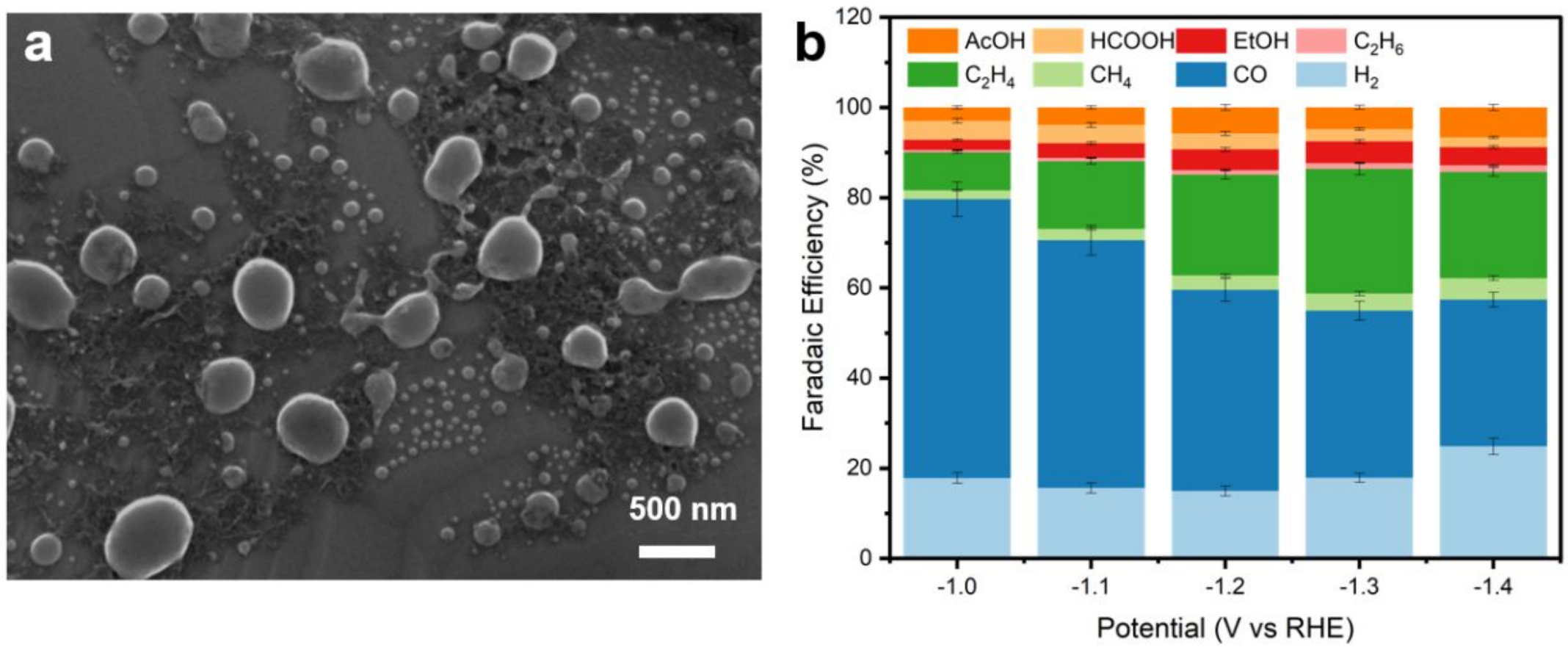

Figure S6. Morphology and CO_2 electroreduction performance of Te-modified non-facet-controlled Cu/Ag. (a) Representative SEM image showing Cu nanoparticles without well-defined THH or HOH morphologies after Te modification. (b) Potential-dependent product distributions of the Te-modified non-facet-controlled Cu/Ag catalyst from −1.0 to −1.4 V. Error bars represent the standard deviation from 3 independent measurements.

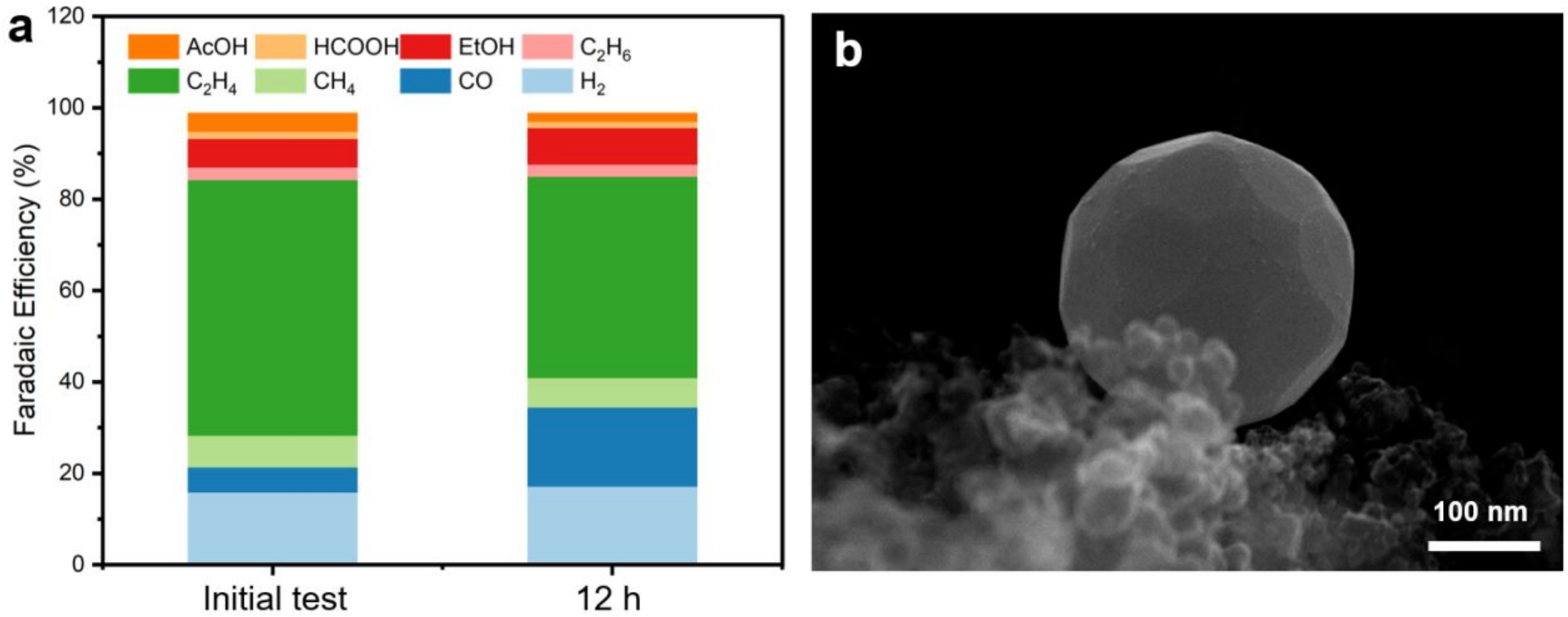

Figure S7. (a) Product distributions measured at the beginning of the stability test and after 12 h of continuous operation at −1.4 V. (b) Representative SEM image of the catalyst after the 12 h test.

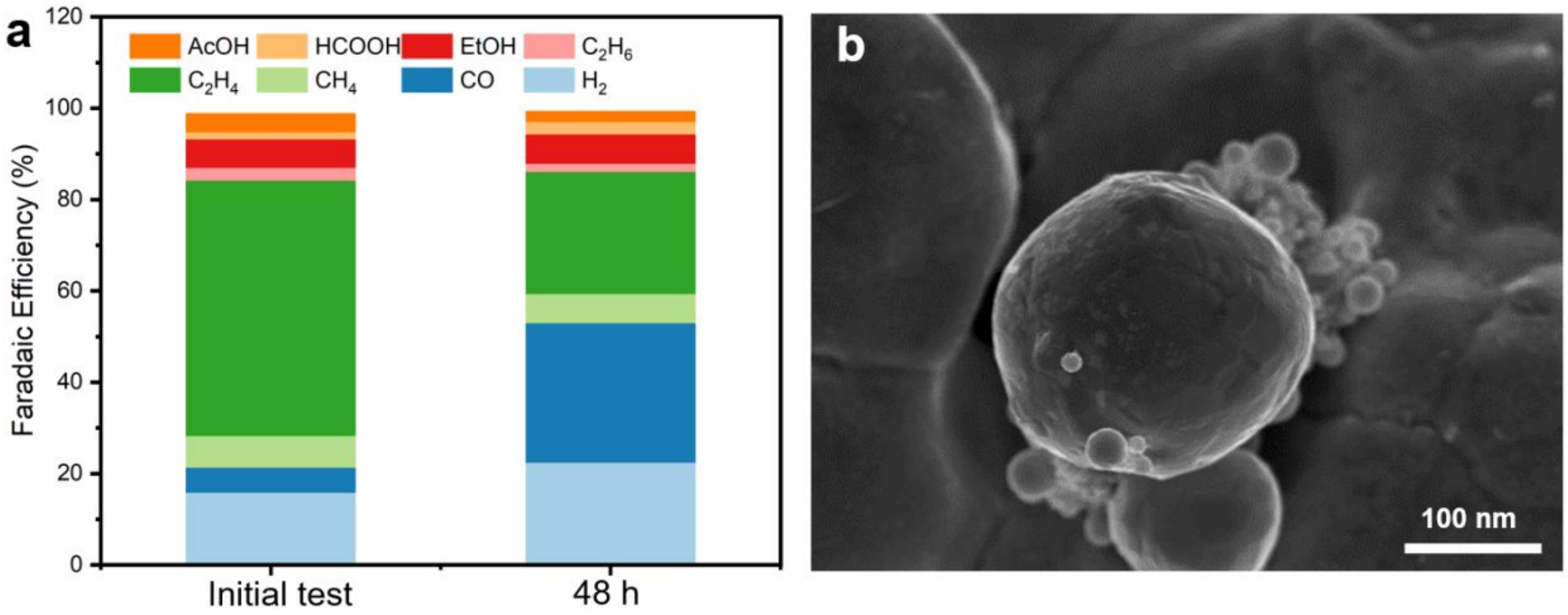

Figure S8. (a) Product distributions measured at the beginning of the stability test and after 48 h of continuous operation at −1.4 V. (b) Representative SEM image of the catalyst after the 48 h test.
